# On drug discovery against infectious diseases and academic medicinal chemistry contributions

**DOI:** 10.3762/bjoc.18.141

**Published:** 2022-09-29

**Authors:** Yves L Janin

**Affiliations:** 1 Structure et Instabilité des Génomes (StrInG), Muséum National d'Histoire Naturelle, INSERM, CNRS, Alliance Sorbonne Université, 75005 Paris, France

**Keywords:** antibacterials, frequent hitters, hit to lead chemical library, medicinal chemistry, virtual docking

## Abstract

This perspective is an attempt to document the problems that medicinal chemists are facing in drug discovery. It is also trying to identify relevant/possible, research areas in which academics can have an impact and should thus be the subject of grant calls. Accordingly, it describes how hit discovery happens, how compounds to be screened are selected from available chemicals and the possible reasons for the recurrent paucity of useful/exploitable results reported. This is followed by the successful hit to lead stories leading to recent and original antibacterials which are, or about to be, used in human medicine. Then, illustrated considerations and suggestions are made on the possible inputs of academic medicinal chemists. This starts with the observation that discovering a “good” hit in the course of a screening campaign still rely on a lot of luck – which is within the reach of academics –, that the hit to lead process requires a lot of chemistry and that if public–private partnerships can be important throughout these stages, they are absolute requirements for clinical trials. Concerning suggestions to improve the current hit success rate, one academic input in organic chemistry would be to identify new and pertinent chemical space, design synthetic accesses to reach these and prepare the corresponding chemical libraries. Concerning hit to lead programs on a given target, if no new hits are available, previously reported leads along with new structural data can be pertinent starting points to design, prepare and assay original analogues. In conclusion, this text is an actual plea illustrating that, in many countries, academic research in medicinal chemistry should be more funded, especially in the therapeutic area neglected by the industry. At the least, such funds would provide the intensive to secure series of hopefully relevant chemical entities which appears to often lack when considering the results of academic as well as industrial screening campaigns.

## Introduction

### The current state of affairs in the academia and two problems for medicinal chemists

Across the world, medicinal chemistry is often not being considered as a true academic research domain. In many countries, the decision to delegate this aspect of drug discovery to the industry was instrumental in the policy choices made by their public funding agencies. However, at least in the research domains of anti-infectious or neglected diseases, such choices appear to have impacted the actual number of drugs discovered in these countries and this trend goes beyond the academic contributions of the considered country. Looking at the actual origins of the research entities who provided the anti-HIV1 drugs available today is probably the hardest evidence of the consequences of such choices. Moreover, the recent deluge of reports describing the results of virtual screening and meaningless drug repurposing to address the COVID-19 epidemic is, in far too many instances, only symptomatic of a loss of medicinal chemistry culture caused by this lack of academic support. Indeed, virtual docking has yet to demonstrate that it was instrumental in preselecting a really successful hit out of chemical libraries and considering, for instance, anticancer drugs as potential antivirals is barely more relevant than assaying curcumin or (iso)quercetin, not to mention hydroxychloroquine. Past these rather scathing comments which could lead to a long-delayed but healthy debate in the concerned countries, the last four decades saw several “revolutions” in medicinal chemistry which were triggered by the following major advance in sciences:

– The development of genetics and molecular biology leading, with or without the use of chemical probes [1], to the discovery of a near infinite number of biochemical processes, amenable to the design of assays as plausible targets for drug discovery programs.

– The development of robotics and miniaturization to undertake fast screening campaigns of very large chemical libraries using targets and/or phenotypic-based assays.

– The development of structural sciences providing very precise ideas on the kinetic, thermodynamic or on the mode of interactions of chemicals with their biochemical targets. Which, amongst many other uses, provided the background for fragment-based drug design [2–3].

– An ever-increasing computer processing speed leading to an ever-growing list of software-based approaches to try to help in various aspects of drugs discovery. The neural network-based software AlphaFold [4], which is providing a very large database of predicted protein structures, being one of the latest achievements in the domain [5].

– The development of organic chemistry tools enabling a faster synthesis of compounds and, more important, a faster purification/identification (i.e., DNA-tracked synthesis, parallel/combinatorial synthesis, multicomponent chemistry, metal-catalyzed coupling reactions as well as NMR, preparative/analytical liquid chromatography and mass spectrometry).

– An improved understanding of molecular pharmacology along with in vitro or in vivo assays to detect various aspects of drug metabolism and pharmacokinetics which will lead to unacceptable side effects in clinical trials [6].

However, despite all these substantial improvements, the discovery of a chemical that makes it to a use in human medicine remains a really hard thing to achieve. Even at a more modest level, securing a selective effect on an animal model of a given disease is still not an easy goal. This has led to extensive questioning [7–21], including a call for a better teaching of medicinal chemistry in academia [22]. The following plea will certainly not provide instant solutions to such a complex and multidisciplinary process [23] but, through illustrations of the issues in which organic chemistry is involved, it is an attempt to provide a background for academics to focus on. Moreover, many fine textbooks on medicinal chemistry should also be consulted [24–32]. As organic chemists, the main task at hand in a multidisciplinary drug discovery program starts with the initial problem of securing a compound endowed with a biological effect of interest: a “hit”. Then, the second problem arises as the program will require a “hit to lead” process involving many iterations of design/synthesis and biological assessments using a progressively growing list of assays [33]. If successful, the resulting “lead” is usually a class of structurally related compounds compatible with human pharmacology and demonstrating, at least, a tangible effect on an animal model of the disease. Then, past these two hurdles, the task of selecting a clinical candidate and produce up to tons of it is also a major endeavor that has been often overlooked by the academia. However, a noteworthy exception would be the recent flow chemistry developments in the pharmaceutical industry which certainly owe some of its origin to the academic world [34–37]. This perspective will not describe the true chemical challenges as well as the many environmental issues surrounding this last problem. All these are actually well described in books [38–43]. This text only focuses on what happens chemistry-wise during the first two phases: hit discovery and hit to lead, since these are so far remaining within the realm of academia.

## Perspective

### Hit discovery, frequent hitters and virtual screening contributions

**Figure 1:**
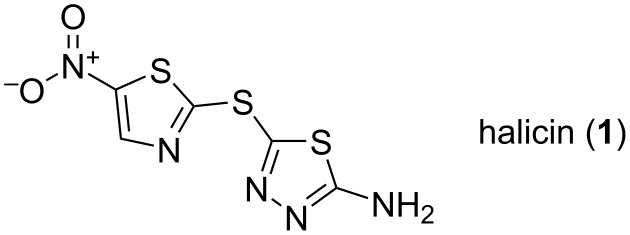
Structure of halicin (**1**).

The first problem is that the discovery of a drug always revolves around a selection of the initial hits to work on. Aside from being “real hits” and not one of the many frequent hitters/pan-assay interference compounds (PAINS), the choice of such compounds is dependent on the chemical libraries available, on the chemistry possible, on the precedents in the literature and patents as well as on the past experience of the chemists involved. In other words, from a bad hit nothing will come out. The whole difficulty is to define a bad hit. Aside from a growing list of “obvious” ones [44–47], the definition of a bad hit is chemist [48–50] and chemistry-dependent. Indeed, if one can, with the right chemistry and insights, quite often provided by structural sciences, reach the really active and selective analogue then it was not a bad starting hit. Concerning the obviously useless hits, these can often be detected by proper control experiments following the screening of a library [51–52]. However, many of these compounds, often of natural origin [53]; polyphenols and curcumin being emblematic [54], are the bread and butter of quack medicine. Indeed, these will be found active on many biochemical or cellular assays [46] and will thus comfort whatever ailment cures are claimed. Unfortunately, this belief-based business represents a substantial economic activity. Moreover, in these post-truth times, it is leading to non-scientific behaviors much too well-illustrated in the course of the COVID-19 pandemic. Even worse, there are many instances in which these commercially available “natural nutrition/food complements/traditional plant extracts/nutraceutics” were found to be spiked with copious amount of authorized drugs, sildenafil being a recurrent one, or even experimental substances [55–56]. In any case, frequent hitters of all sorts, such as rhodanines, are plaguing chemical libraries as well as scientific literature or patents. This situation has actually hampered successive generations of computer-based approaches which attempted to gather these data into something exploitable. The sentence “garbage in garbage out” is a real issue in this regard as a major portion of published data will have to be filtered out before such methods starts to make some tangible headways [57]. For instance, a recent “deep-learning” search for new antibiotics came out with the finding that halicin (**1**) depicted in Figure 1 was, as many nitro-bearing substances, endowed with an antibacterial effect [58]. However, this compound was originally patented for a fungicide effect [59] and then found to also inhibit mammalian c-Jun N-terminal kinase [60]. Unsurprisingly, it was also reported in 2022 as a covalent inhibitor for the SARS-CoV-2 main protease [61]. Unfortunately, the inherent reactivity of compound **1** is the reason for these effects and it is likely that many more reports describing a whole array of useless in vitro biological properties will be published in the future.

**Figure 2:**
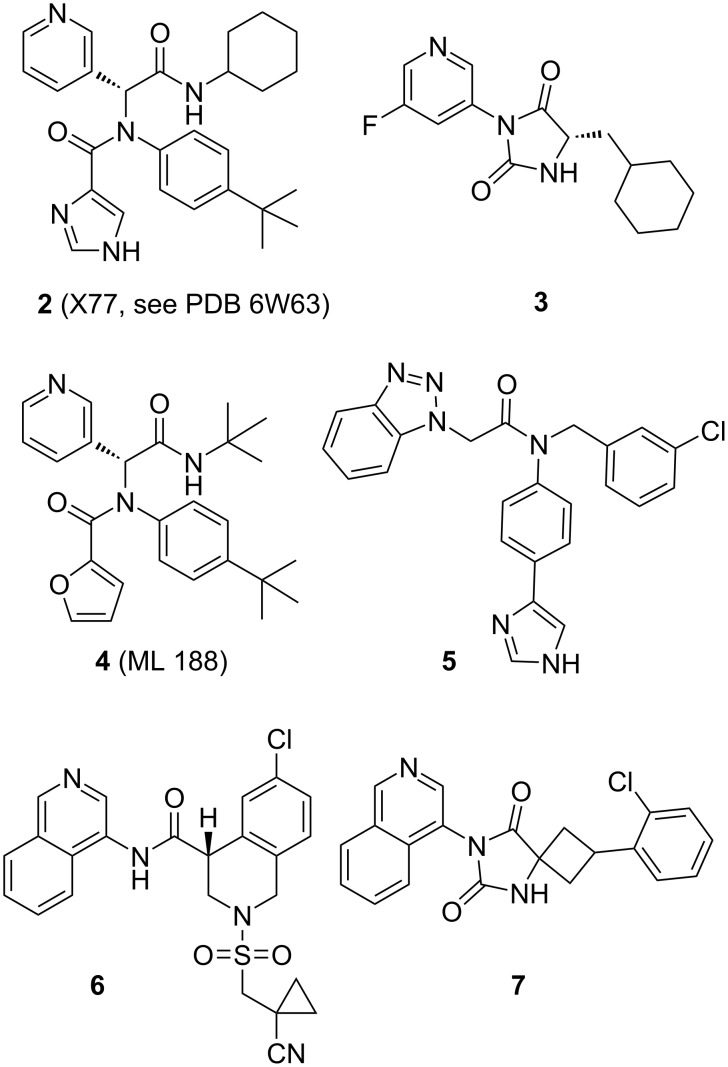
Structures of compounds **2–7**.

Also on this issue, the opening sentence in the abstract of a review [62] focusing on hit selection (triage) is probably a good general starting point to describe the problem which academics are facing: “It is increasingly clear that academic high-throughput screening (HTS) and virtual HTS triage suffers from a lack of scientists trained in the art and science of early drug discovery chemistry.” Indeed, an embarrassingly large number of hits resulting from academic screenings and reported as potentially useful could be used to further illustrate this as well as the corresponding waste of resources [63]. This problem is not new and a *déjà vu* feeling comes to mind when reading a 2010 commentary [64]. In any case, before this latest knowledge-based approach, many computer programs had been written to select compounds out of available chemicals for a biological screening. When well designed, these pre-screenings are very useful to remove many of the obviously bad compounds [65–68]. But past such filters, the many software designed in the last 30 years to predict which chemicals will be active on a given target, still feature a “large room for improvement” [69]. Concerning ligand-based virtual screening also known as hit expansion, this is a really useful approach to identify bioisosteres [70–71] or to undertake scaffold hoping [72] from an actual hit. On the other hand, concerning the virtual docking [73–74] of molecules on structurally characterized targets, past a rather sobering 2010 domesday report [75], a more recent review [76] is noteworthy at the least for the two following quotes: “Due to the inherent inaccuracies of molecular docking, visual inspection of binding modes is a crucial routine in the decision making process of computational medicinal chemists.” and “This suggests that the journey to reliable scoring functions is by far not over, as today’s scoring functions are often no match for the complex knowledge and vast experience of computational medicinal chemists.”. In other words, virtually screening millions of compounds appears to still require a completely unrealistic visual checking of each docking solutions. Another recent survey on the efficiency of various programs is also worth consulting as it appears that close to half the docking solutions provided in the context of COVID-19 research for antivirals were useless to guide medicinal chemistry efforts [77]. A very recent illustration of the difference of actual results secured with either a virtual docking approach or a ligand-based search would be the ultra large virtual screening of 235 million compounds which was undertaken against SARS-CoV-2 main protease [78]. As depicted in Figure 2, this was undertaken using the X-ray based structure 6W63 which in facts features the virus protease binding the non-covalent inhibitor X77 (**2**). Of importance is that this compound derives from inhibitors of SARS-CoV-1 main protease, such as **4** and **5**, which were found in 2013 [79–80]. Interestingly, this truly large virtual screening came out with (only) three validated hits, including the 3-pyridyl bearing hydantoin **3**. However, the same research group also performed a “fragment-guided virtual screening” using biological and structural data all based on the previously known inhibitors **4** and **5**. As for the remarkable COVID Moonshot initiative, it is these 3-pyridyl- or benzotriazol-containing hits which were the crucial starting points to independently reach promising corona viruses main protease inhibitors such as compounds **6** and **7**. And this was only achieved following many iterations of structure-based design, synthesis and assays of analogues [78,81–82]. Also of concern, a subsequent virtual hit expansion/ligand-based screening starting with the isoquinoline-bearing inhibitors such as **6** and using the Ukrainian REAL database which contains 1.37 billion of compounds, actually failed to go beyond this chemical motif [83]. In any case, what would have been the results of all these computer-based endeavors had the hits **4** and **5**, which led to compounds **6** and **7**, not been reported [79–80] in 2013? In fact, it is a well-designed and quite “blind” screening of 293.000 compounds from the NIH molecular libraries [79–80], luckily containing such fruitful hits, which saved the day almost a decade later!

For another very recent illustration of the risks of only relying on virtual docking for drug discovery, a reported attempt to design peptidic but non-covalent inhibitors of SARS-CoV-2 main protease is also worth consulting [84]. To tone down this sometime sobering paragraph, the very recently published practical guide for large-scale docking must be mentioned here as it should, at the least, lead to a general practice improvement [85]. Concerning which kind of computer-predictable or known criteria are used today to select compounds prior undertaking a real screening, current trends are found in reports from the industry. A paper from Novartis describes that solubility and cell permeability are, for phenotype-based screenings, the favorite criteria along with structural diversity in regard with previously observed biological effects [86]. Along with such parameters, a Bayer approach is also aiming at increasing their chemical libraries by half a million compounds in the next five years in order to address a “novelty erosion” [87]. This means that the chemical libraries we can have today do not encompass the chemical space of drug-like compounds, far from it, apparently [88–91]. Concerning the definition of drug-like compounds, past the ground breaking Lipinski’s rule of five [92], defining more precise criteria for the inclusion of a given chemical, possibly dependent on the target considered, remains an interesting research subject [88,93–96]. A survey of the evolution of commercially available chemical libraries is also of real interest [97]. It turns out that a low lipophilicity, a higher fraction of sp3 carbons [98] in the structures as well as a minimum of 20 compounds per scaffold are currently favored. A high sp3 proportion along with a structural diversity are also prominent factors chosen for the ESCulab library made available by the European Lead Factory initiative [99]. Concerning this minimum of related compounds to include in an assay, it is likely stemming from Ulf Norinder’s probability-based illustration of the issues surrounding chemicals selection from libraries at Astra Zeneca. In his demonstration he is considering a rather too large one million compounds library in which three singletons hits for a given screening are present [30]. The term singleton is conveying here that there are no structurally related and active analogues present in this library. If a screening of the full set will ensure their discovery, shrinking the library to a more manageable 150.000 compounds leads to a 0.29% odd of finding these three distinct singletons. On the other hand, had the original library contained several active compounds structurally related to these singletons, the odds to discover them using a subset of the library would have improved. However, this comes with the problem of losing some of the overall chemical library diversity. Thus, any type of computer-based selection of compounds, especially in the context of modestly funded screening programs, is very likely to miss singleton hits. Since this point is relevant whatever the size of the chemical library to be screened, serendipity remains a key requirement for any successful hit discovery. Similar considerations are probably what led a Wyeth-Ayerst research group [100] to retain between 50 and 100 compounds for each series within a chemical library in order to improve the “hit-rate within a series”. Our discovery of an original inhibitor of human dihydroorotic acid dehydrogenase [101], certainly owe its modest success to this, since the entire library, including a crucial methylated derivative, was assayed [102–103]. This methyl effect, which addition on the structure of a compound is essential to detect a biological effect within the considered series, is also seen past the screening stage. Indeed, many successful drugs were obtained following a methyl incorporation in the course of attempts to improve the medicinal chemistry of the parent structure [104]. A recent review is actually listing the wide range of reasons accounting for this fact as well as the recent chemistry which has been developed to “plant” such methyl groups on a given compound [105]. In fact, the terms *planteurs de méthyle* (methyl farmers) was routinely used in the 1990s to name medicinal chemists in a major French pharmaceutical company. The replacement of a CH with a nitrogen in a ring system can have similar improvement effects [106–107] although the expression *carbon transmuter* has yet to be used in MedChem to name chemists undertaking such positional scanning strategy. Still on the singleton subject, a hit generation group was created at Janssen to “rescue some projects” starting from such isolated hits. This group soon mushroomed into a multidisciplinary team capable of dealing with many aspects of medicinal chemistry such as “new screening paradigms, computational approaches, novel synthetic chemistry, gene family screening, investigating routes of delivery and so on” [108].

### Hit to lead, recent success stories in antibacterials

Past this essential point, which again does govern the eventual success of the whole drug discovery process, the second problem is also a real issue. In a world where millions of chemicals can be subjected to a biological screening in few months [109], the ensuing iterations of design/synthesis/evaluations to improve and adapt the validated hits to human/mammalian pharmacology are comparatively and inherently very-very slow. The time needed for the preparation of a handful of analogues of a hit will depend on the chemistry and the number of synthetic steps required. When the chemistry is neither known nor easy, this can take up to months of work. Then, the necessary feedback from the assays along with quite a few controls and further evaluations (i.e., from ligand binding thermodynamics, if the target is known, to cellular toxicity and all the way to early ADME) will also take some time. In other words, organic synthesis remains one of the limiting factors in drug discovery since it is a rather handcrafted job requiring a lot of qualified staff to prepare up to thousands of analogues of a hit and there is still no guarantee of success. On this issue, a very thorough comment written by Derek Lowe on his excellent *in the pipeline* blog [110] is worth reading in full. Moreover, applying too much pressure on the chemists to produce these analogues, or subcontracting the whole process, is likely to orient the synthetic work toward the preparation of “easy” compounds. The inherent risk then being to miss the initially hard to get but way better (rescaffolded?) inhibitors which would have taken the market 10–15 years later. The following is an attempt to illustrate the issues encountered in this second stage with a review of the new antibiotics discovered which are, or about to be, used in human medicine.

**Figure 3:**
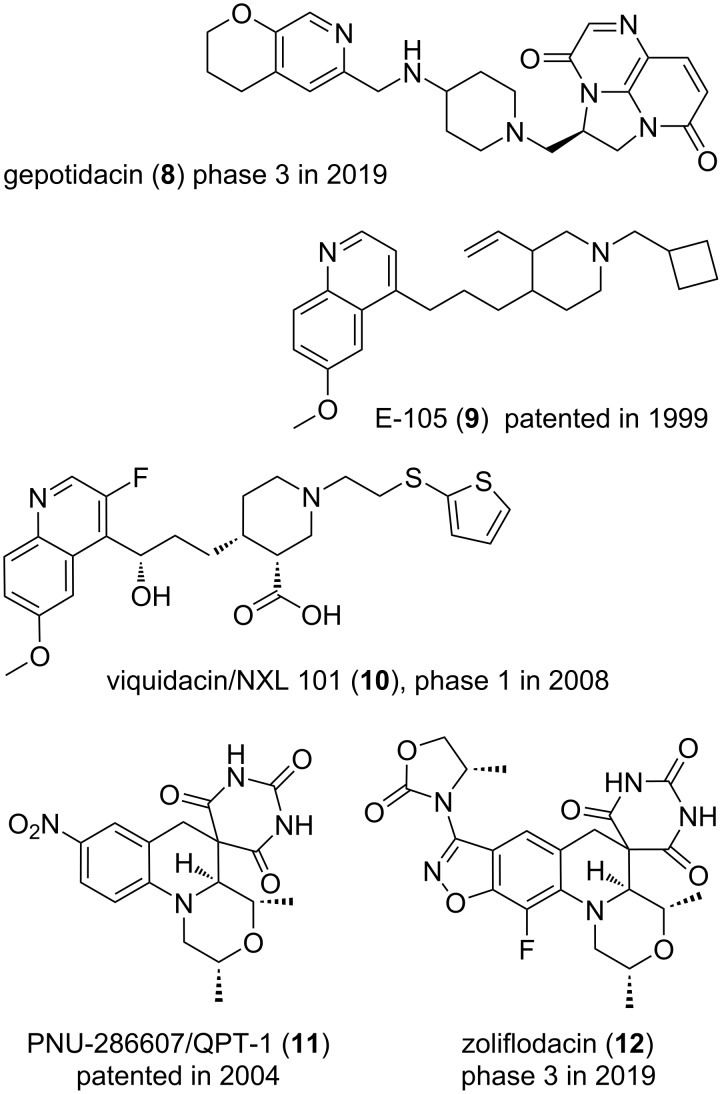
Structures of compounds **8–12**.

In 2007, the antibacterial research department of GSK reported the results of their genome-driven seven-year long quest for original antibiotics. This target-based approach actually failed although 300 bacterial genes had been considered and 70 high-throughput screening campaigns, focusing on the corresponding proteins, had been undertaken. Following this terribly sobering fact, plausibly often due to bacterial efflux pumps [111], the resulting strategic decision was a serious increase of the proportion of chemists working on a given research project as well as a refocus on chemicals acting on established bacterial targets [112]. History will judge the long-term wisdom of this choice although GSK has already reported gepotidacin (**8**) an antibiotic currently in phase 3 clinical trials [113–114]. Of importance is that, as depicted in Figure 3, the starting point of this new class of bacterial gyrases inhibitors [115] was a SmithKline Beecham/GSK patent which disclosed in 1999 the effect on the growth of Gram-negative and Gram-positive bacteria of compounds such as E-105 (**9**) [116]. Extensive hit to lead programs were undertaken in quite a few pharmaceutical companies and in 2008 Novexel, a spinoff of Aventis/Sanofi, sponsored the phase 1 clinical trial of NXL 101 (**10**) [117]. This trial was however quickly stopped because of a forbidding QTc prolongation seen in human cardiograms. It then took a lot more synthetic work, illustrated by over 100 distinct patents, to alleviate this hERG channel-related cardiotoxicity risk and reach, in 2019, gepotidacin (**8**) a first in class bacterial gyrase inhibitor. Moreover, the design of original analogues is still going on as illustrated by recent reports [118–121]. Another proof of the necessity of allowing time for the maturation of series of compounds would be a second original class of bacterial gyrases inhibitors. In 2004, the nitro-bearing derivative **11**, resulting from a high-throughput phenotypic-based screening, was patented by Pharmacia/Pfizer for its antibacterial properties [122–123]. Even if this compound was also effective in vivo, many synthesis and evaluation iterations were undertaken at Astra Zeneca to reach, in 2014, zoliflodacin (**12**) [124] which is currently undergoing a phase 3 clinical trial, sponsored by Entasis Therapeutics [125]. Interestingly, further synthetic work is going on [126–127], including on original analogues active in vivo against *Mycobacterium tuberculosis* [128]. Of note is that in 2011, Astra Zeneca undertook some rather extensive strategic changes which led to an improvement of the drug discovery productivity and, possibly, to zoliflodacin (**12**) [129].

**Figure 4:**
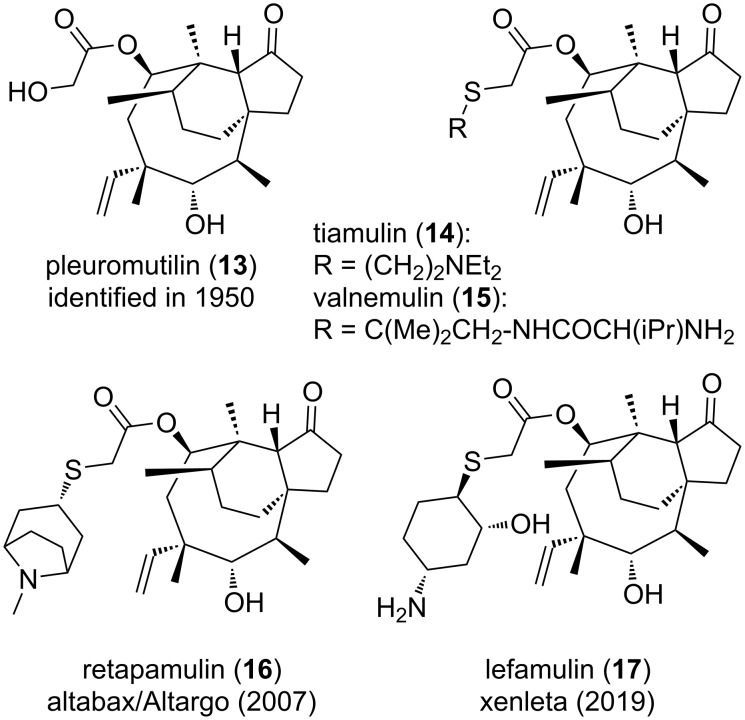
Structures of compounds **13–17**.

Another class of antibiotics deserving a place here should be the derivatives of pleuromutilin (**13**) depicted in Figure 4, which were developed across 70 years, initially for veterinary and then human medicine [130–133]. This naturally occurring inhibitor of bacterial ribosome [134] was discovered [135] in 1951 and the two hemisynthetic analogues tiamulin (**14**) and valnemulin (**15**) were then developed to fight swine dysentery or respiratory infections [136–139]. In human medicine, if retapamulin (**16**) was found to be effective topically against human skin infections [140], it then took more years to reach the orally active and remarkably simple-looking analog lefamulin (**17**). Indeed, this compound was first prepared at Nabriva in 2006 [141–142], and was approved for human use in 2019 [143–144]. The still ongoing story [145] of pleuromutilins illustrates again how long a successful discovery process can take. Concerning one last class of large spectrum antibiotics, there are noteworthy reports which describe the extensive rescaffolding efforts aiming at the discovery of original fluoroquinolone-like antibiotics [146–148].

**Figure 5:**
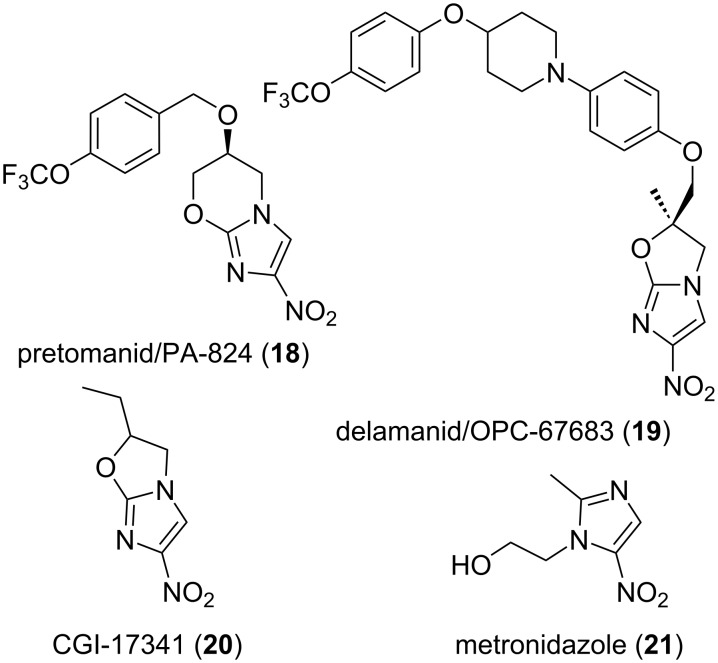
Structures of compounds **18–21**.

The recently reviewed [149] discoveries of the bicyclic nitroimidazoles depicted in Figure 5: pretomanid (**18**) and delamanid (**19**) as new drugs to treat tuberculosis is also of interest. The starting point was probably CGI-17341 (**20**), a mutagenic compound reported by Ciba-Geigy in 1989 for its effect on mycobacteria [150]. And this substance owes its origin to the many nitroimidazole derivatives known for their anti-infective effects such as metronidazole (**21**), which is still used to treat anaerobic infections [151]. In any case, pretomanid (**18**), first mentioned in 1996 and reported [152] for its effect on mycobacteria growth in 2000, was developed by the TB alliance and approved for use in human in 2019. Again, this was the result of extensive structure–activity relationship studies, especially to avoid mutagenic effects, undertaken by PathoGenesis/Novartis. Similarly, delamanid (**19**), first reported [153–154] in 2006 and approved in 2014 for its use against tuberculosis, was the fruit of extensive research made by Otsuka, which included the synthesis of close to 3400 analogues. Moreover, further synthetic work is ongoing since this class of anti-infectious agents may have a use against kinetoplastid protozoan parasites to treat neglected diseases such as African trypanosomiasis or leishmaniasis [149,155].

**Figure 6:**
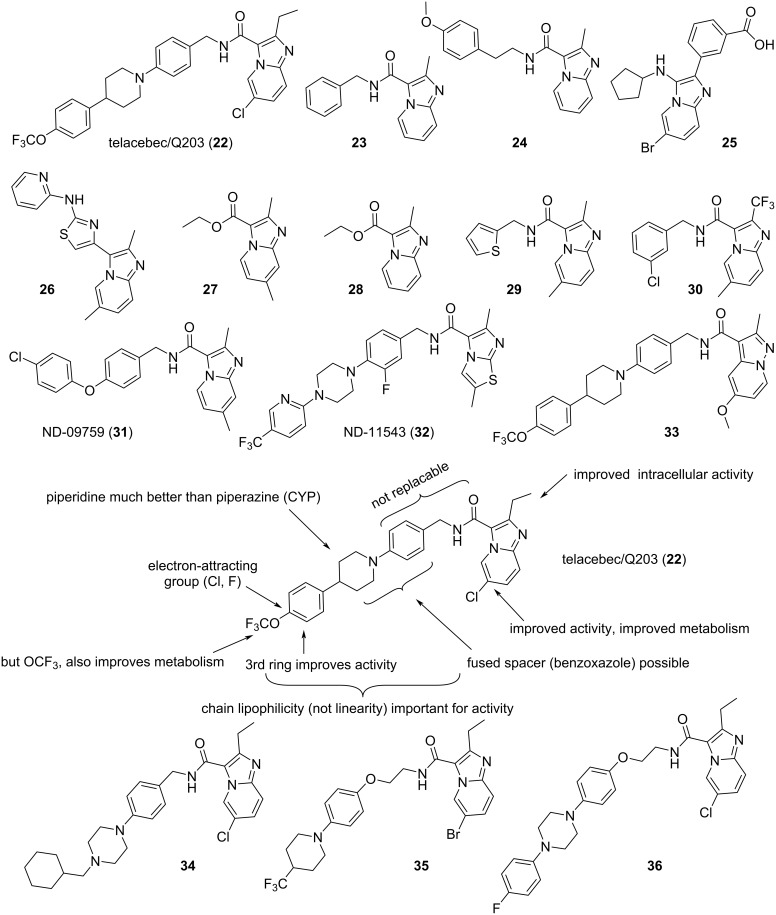
Structures of compounds **22–36** and SAR of compound **22**.

As depicted in Figure 6 and still on mycobacterial infection [156], one last example would be telacebec/Q203 (**22**) a compound which, following a patent [157] filed by the Korean Institut Pasteur in 2010, underwent in 2020 a successful phase 2 clinical trial, sponsored by Qurient, for its use against tuberculosis [158]. The initial hits came from a phenotypic screening which, out of more than 120,000 molecules, found the antimycobacterial effects of imidazopyridines **23** and **24** [159]. It would be of interest to retrace the reason for the presence of these two compounds in the chemicals screened, random luck seems the most likely factor, although other screenings for antimycobacterials reported the imidazopyridine derivative **26** as well as the ester **27** around the same time [160–161]. Concerning ester **27**, it actually came from a DowAgroScience chemical library although, as early as 2004, the closely related compound **28** (note the missing methyl group) had been reported to be devoid of antimycobacterial effects at 6.25 µg/mL (30 µM) [162]. Moreover, a screening at GSK identified the amides **29** and **30** [163–164], and amine **25** was also reported but only for an effect on *M. tuberculosis* glutamine synthetase [165]. Interestingly, aside from compounds **23** and **24** (MIC_50_ = 1.9 and 2.6 µM), the more modest activity of ester **27** (MIC_90_ = 65 µM) also initiated a hit to lead research program [161,166–169], resulting in a first patent filed [170] in 2009 and for instance, ND-09759 (**31**) an antimycobacterial active in vivo [171] as well as the rescaffolded analogue ND-11543 (**32**) [168–169]. In view of this, further scaffold-hopping was undertaken [172–173], as illustrated by the structure of the pyridopyrazole **33** which is also featuring the long aryl-bearing chain of telacebec (**22**) [174]. Concerning the structure–activity and structure–pharmacology iterations leading to telacebec (**22**) [157,175–177], innovation in the design, synthesis and evaluations of 477 analogues (by 13 chemists) was again essential [159]. Luckily, these analogues could address the various problems encountered in the course of the hit and then lead optimizations. As depicted, many structural components of this class of drug were found to be of importance and such results cannot be secured without a lot of iterations.

Moreover, additional work conducted in another research laboratory also led to good inhibitors such as the cyclohexyl-bearing compound **34** [178] as well as the amidoethyl ether-bearing derivatives **35** and **36** [178–180]. Finally, a data base search on the sole amido-imidazopyridine component of this class of compounds came out with 1,500 derivatives which demonstrates that this ring system is still the focus of investigations. Some of these have led to deuterated analogues of these antimycobacterials [181–182] or to recognize that these compounds have also an effect on human arachidonate 5-lipoxygenase and are thus potentially useful as anti-inflammatory agents [183].

### Suggestions to improve the output of academic drug discovery programs

#### Foreword, matchmaking a good compound with a proper screen, can academics do this?

**Figure 7:**
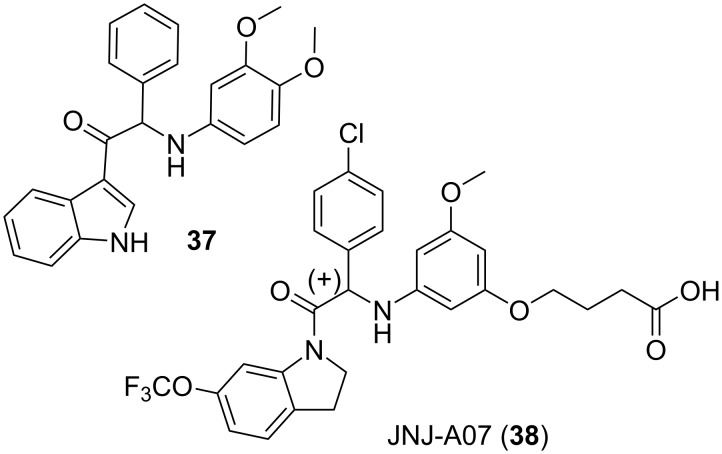
Structures of compounds **37** and **38**.

Remarkably [184], the discovery of all these original antibiotics started with phenotypic-based screenings of the right chemical libraries and was followed by extensive structure–activity relationship studies involving a large array of assays. Of course, organizing and funding these endeavors required considerable investments as well as the gathering of experts in many research domains. Interestingly, the last few decades saw the emergence of a lot of contract research organization (CRO) providing for most, if not all, the know-how required for drug discovery [185]. Many companies, usually starting with the design of an original screening (virtual/target or phenotypic-based), made extensive uses of these CRO in attempts to discover new drugs while avoiding some costs. It would be out of the scope of this perspective as well as the experience of this author to try to draw conclusions on the relative success rate of this business model [186]. However, the availability of these CRO do provide the required services which would have been beyond the reach of most academics attempting to undertake a drug discovery project. This means that an academic consortium, gathering a core of scientific expertise, can, starting from initial results of potential interest, credibly apply to grant calls aiming at a drug discovery. For this reason, a key point of this whole text is a plea to include in these consortium academic chemists who can also be important sources of intellectual property. The European Lead Factory facilities [99,187–189], the infection innovation consortium (IICON) [190] or the now decade-old GSK initiative at the Tres Cantos open lab [191], as well as many NIH initiatives such as the Antiviral Drug Discovery Centers [192] are also available for such projects. The well-illustrated perspectives on the organization of such public private partnerships, often focusing on neglected diseases, are worth consulting [193–197] as well as a recent survey on the contributions of private and public sector to biopharmaceutical research [198]. The recently reported results of a medium throughput phenotypic-based screening of “many thousands of compounds” for chemicals active against the dengue virus would be a very good illustration of the potential of such partnerships [199–200]. In this case, as depicted in Figure 7, from the hit **37** found, the ensuing structure–activity relationship studies of 2000 analogues led to JNJ-A07 (**38**) which has between nanomolar and picomolar level of effects against dengue virus replication in cell lines and as well as an in vivo effect on a mice model of infection. Of note is that the hit to lead process was undertaken by the Centre for Drug Design and Discovery and it resulted in a portfolio of patents assigned to Janssen and the catholic university of Leuven [201–203]. Interestingly, these patents are protecting a truly original class of antivirals which solely target the dengue virus via the inhibition of an essential interaction between the viral proteins NS3 and NS4B [200].

**Figure 8:**
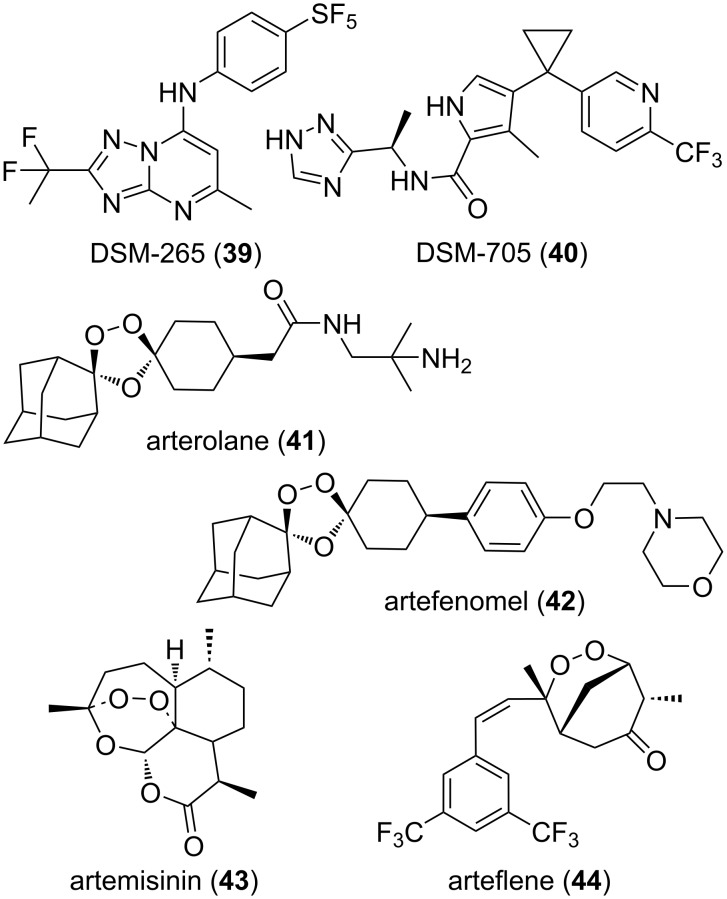
Structures of compounds **39–44**.

Still in the domain of infectious diseases, there are two very advanced public-based endeavors aiming at providing drugs against malaria which are illustrated in Figure 8. The still ongoing quest for drugs acting on *Plasmodium* dihydroorotate dehydrogenase was initiated around year 2000 and gathered academic groups across the world. This has resulted, in 2018, in a phase 2 clinical trial [204] of the inhibitor DSM (Dallas-Seattle-Melbourne)-265 (**39**) [205–207] and more recently the discovery of a backup series which is illustrated by the in vivo effect of DSM-705 (**40**) [208–209]. It is important to mention that the starting point of these results were in both cases hits found in the course of large scale and target-based screenings of available chemical libraries. With a very different starting point but also on the malaria front, the remarkable ozonides OZ277/arterolane (**41**) [210–211] and OZ439/artefenomel (**42**) [212–214] stemmed from academic collaborations across the world which started in 2000. While arterolane (**41**) is a drug used in combination with piperaquine (Synriam), the analog artefenomel (**42**) is undergoing clinical trials, including in combination with DSM-265 (**39**) [215–218]. Of note is that prior to 1971, thinking of developing a peroxide-containing compound for a clinical use against malaria would have not been considered relevant. However, this changed after the identification [219–220] of the temperature-sensitive and endoperoxide-containing qinghaosu/artemisinin (**43**) as the active constituent of the herb *Artemisia annua* to treat fever and malaria in China. For this discovery, the chemist Tu Youyou was granted the 2015 Nobel price. This also triggered extensive research aiming at improving the rather poor pharmacological properties of artemisinin (**43**) and led to many hemisynthetic derivatives [221]. Moreover, artificial peroxide-containing compounds were also investigated at least partly to address the issues of availability and relative scarcity [222] of this natural substance [223–224]. As early as 1994, this resulted in phase 1 clinical trials of the orally available Ro 42-1611/arteflene (**44**) [225]. However, its development was stopped because of the projected cost for a necessary combination therapy [221,224]. It then took quite a while to balance in this class of antimalarial a requirement for a reactive component with an acceptable human pharmacological profile. This was reached with the ozonides **41** and **42** which, in lights of the perpetual selection of drug-resistant *Plasmodium* strains, will have to be used in combination therapies [210–214].

Aside from participating in a multidisciplinary drug discovery program based on the screenings of chemical libraries followed by many rounds of design, synthesis and evaluation of analogues, what other kind of projects could chemists consider? Grant-wise, the last 40 years have seen the rise of chemical biology and the discovery of many types of large constructs providing tools often useful for life science but far less effective in human medicine. An excellent review [226] has recently listed these approaches but even if they have to some degree improved the arsenal of compounds available for human medicine, the inherent pharmacological limitations of these large chemical constructs remains a major issue [227]. This means that for most health problems, “classic” medicinal chemistry is not going to be replaced by chemical biology. To contribute to medicinal chemistry, one axis remains to provide chemical libraries with new chemical entities, another would be to reconsider previous leads and attempt to improve them. A concern for the first axis is that securing a grant for the synthesis of series of drug-like molecules, with the possibility that some may turn active in a screening years after, may be at odd with current academic funding policies. However, when considering past successes, this approach is indeed what provided many compounds, sometime ages before the screenings which led to their discovery. The reasons for their synthesis were often due to preceding hit to lead research programs followed by their inclusion in chemical libraries. But there was also the more academic stance “we can reach these compounds with our new chemistry so let’s investigate its scope and prepare some derivatives”. And all this is what provided the content of a many chemical libraries. In fact, Louis Pasteur’s 1853 report on the sulfuric acid ring opening of cinchonine into cinchonicine/cinchotoxine as well as quinine into quinicine/quinotoxine is what eventually led to the antibacterials **8**–**10** depicted above [228–231]. Across the last 40 years, to address the fundamental issue of accessing new chemical entities, the industry expanded their collections by buying other pharmaceutical companies or via the synthesis/purchase of chemical libraries prepared for instance by combinatorial chemistry. If, initially, the combinatorial approach [232–233] appeared to have only led to one anticancer drug [234], major progresses in the design, selection, generation and purification of libraries have since then turned this type of chemistry into a central tool for drug discovery [235–237] along with more recent success stories [238]. To illustrate the long term benefits of the constitution of chemical libraries and as depicted in Figure 2, it is a 2013 screening on SARS-CoV-1 main protease [79–80] of compounds produced by multicomponent chemistry [239–242], which provided hits such as compounds **4** and **5**. These turned out to also be effective against SARS-CoV-2 main protease [243] and thus provided the key structure-based data for two distinct fruitful hit to lead programs against this virus [78,81–82]. The current generation of DNA-encoded libraries, a technique which stemmed from an academic [244–245] thought experiment and was quickly adopted by industrials [246–247], is also a noteworthy incentive for innovation in organic chemistry [248–252]. However, and still on COVID-19, the DNA-encoded chemical library approach which led to yet another aldehyde-bearing compound effective against SARS-Cov-2 main protease remains to be judged as relevant [253]. Indeed, many hard won lessons learned in the earlier days on the selection of components for chemical libraries generation should be kept in mind [68]. In any case, the content of these libraries is also a matter of chemistry and the following suggestions are only additions to the far more thorough reviews describing recent contribution of organic synthesis to drug discovery [254–257]. A report [258] surveying the most used reactions in medicinal chemistry is also of relevance; although whether these emerged because of chemists’ choices [259–260] or because of the biological effects of the resulting compounds remain an open question when considering the infinity of chemical space. On this question, a quote from a review [57] on data bases mining is of relevance: “[…] medicinal chemists, who have been characterized, we think incorrectly, as conservative because they often tend to use and reuse the same chemical motifs in the compounds they make. Rather, we think this medicinal chemistry behavior is better characterized as pragmatic as professional survival depends on creating compounds to meet project goals, and the use and reuse of chemical motifs previously shown to have useful biological activity are a proven successful strategy”.

#### Provide pertinent series of new chemical entities to chemical libraries

**Scheme 1:**
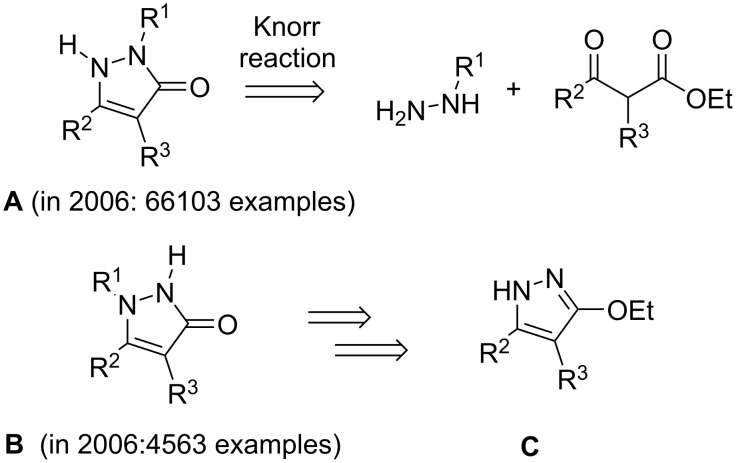
Retrosynthesis of pyrazolones **A** and **B**.

A whole paragraph of a book chapter [261] on the issue of generating new compounds for screening, which somehow echoes with the last quote above, should be mentioned here: “There is a common misconception that “novel” compounds or concepts will lead to dramatically different structural features. It is unlikely that the drug of the future will be built from radically other structural classes than today’s drugs. […] the majority of the compounds will be built from the structural features known already today, yet they may occupy white areas on the map of chemical space”. The future will see if this statement is fully true but as explained above, active singletons in a chemical library appear to be more difficult to discover in a screening, contrary to series of active and structurally related compounds. In other words, when preparing new chemical entities, it may be crucial to synthesize an array of derivatives featuring a variety of substituents and thus cover “a white area” to improve their chances of being identified in the course of screenings. In 2004, the Molecular Library Initiative sponsored by the NIH also tried to address such issues and provide probes to determine the function and therapeutic potential of all the human genes. Aside from considerations on the selectivity and drug-like potential of the chemical libraries produced, it may well be that the policy of discouraging intellectual property claims actually curbed the success rate of this truly massive effort which appears to have stopped in 2015 [262]. In any case, some years ago, we used the image of a “chemical blind spot” to illustrate the existence of such “white area”. When considering pyrazolones with the general formula **A** depicted in Scheme 1, these are very easy to prepare in one step using a Knorr condensation. Whereas, up to 2006, an order of magnitude less of the isomeric derivatives **B** had been reported in patents just because of a lack of a simple synthetic method. Our contribution to alleviate this “blind spot” was an extensive exploration of the chemistry of alkoxypyrazoles (**C**) which provided synthetic building blocks useful to reach, amongst others, many new chemical entities with the general formula **B** [263–271]. Years after their preparation, this strategy led to tools of biological interest once [102–103] and all of them remain in chemical libraries, just waiting to be assayed in a “right” screening.

To pursue this approach, the first step could be to systematically identify promising chemical space, for instance heterocyclic derivatives or heterocyclic systems featuring substitution patterns which have not been prepared yet. Such identification could probably benefit from computer-assisted data mining although this is definitively not the area of expertise of this author. Indeed, can it be possible to assess the “density” of a given known chemical space and identify pertinent and “rarefied/white” areas? Many words in this last sentence could use an extensive definition and a lot of this probably revolves around computer-compatible criteria implied by the word “pertinent”. A report [272] attempting to list all the heterocycles which have yet to be synthetized is of interest in this regard and since then some were prepared [273]. Another paper is also worth consulting as it is listing the many computer-based descriptors which can be used to define chemical space, drug space or activity space [274]. Moreover, an attempt to characterize and expand the “medicinally relevant chemical space” is also instructive on the problems that are encountered, such as a recurrent lack of systematic experimental data [275]. But past these essential points and armed with such information, the use of modern chemical reactions as well as the reinvestigation of old synthetic pathways and the help of modern purification and analytical tools are likely to provide new insights and opportunities to reach new chemical entities. The series of books [276] on heterocycle chemistry edited by Jie Jack Li as well as a recent perspective [277] should also be sources of inspiration. Moreover, aside from heterocycles, there are many other domains of organic chemistry worth exploring. For instance, the large array of conformationally restricted diamines available today is a least in part due to their usefulness in medicinal chemistry [278] and research to reach even more elaborated amines is warranted. Concerning natural product synthesis, which has been the main source of chemical synthesis challenges in the last century, Paul Wender’s approach consisting in also reaching for simplified analogues, hopefully retaining the function of the biologically active compound, is very relevant when considering its potential in medicinal chemistry [279–281].

#### Start from leads obtained from previous research

**Scheme 2:**
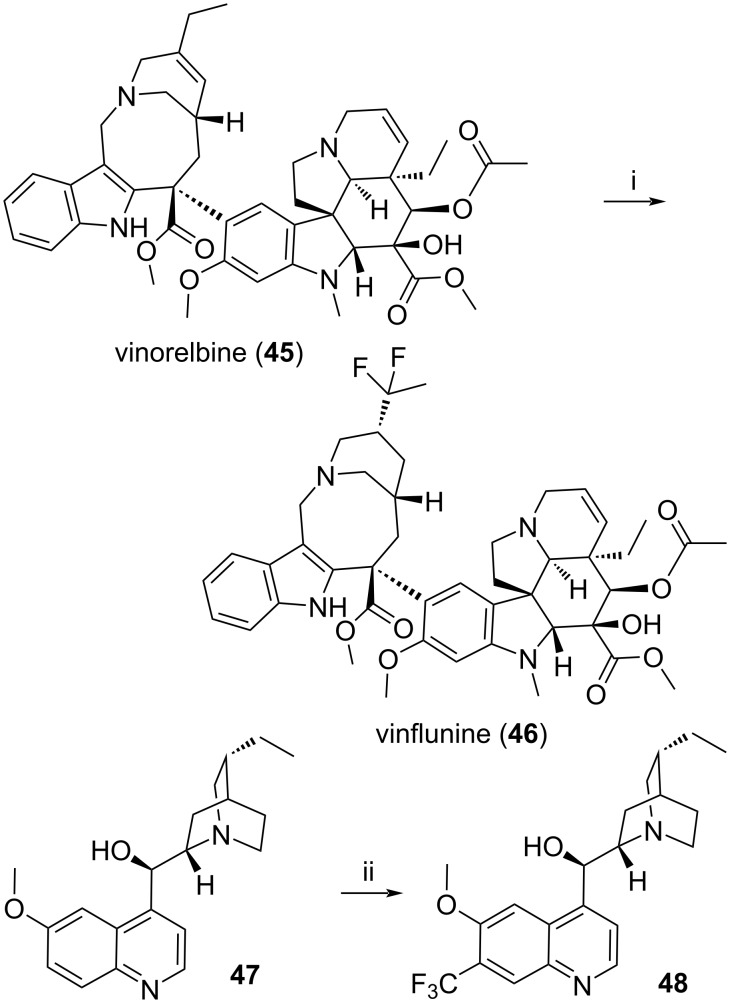
Reaction conditions: i: HF, SbF_6_, CCl_4_. ii: (CF_3_SO_2_)_2_Zn, *t*-BuO_2_H, CH_2_Cl_2_/H_2_O.

A second research direction for chemists is to start from previously published/patented series which are worth some more work and this sometime leads to a “best-in-class” drug as opposed to the “first-in-class” one. This is a well-trodden path [282–285] which has led Sir Arthur James Black, the developer of cimetidine, to state [286] “the most fruitful basis for the discovery of a new drug is to start with an old drug.” A really interesting paper from Genentech/Roche describing the evolution of their approaches between 2009 and 2020 to discover leads is actually pointing out that publicly available data remained their main source of inspiration followed by high-throughput screenings [287]. This strategy is certainly visible when considering compounds **34**–**36** or the switch from artemisinin (**43**) to the peroxides **41**, **42** and **44** depicted above and it is also a source of chemical challenges for instance to achieve a scaffold hoping or a bioisosteric replacements [288–293]. Moreover, the use/design of new chemical reactions may lead to hard-to-get and original analogues possibly better than the one reported. One example, depicted in Scheme 2, would be the fluorination, under superacid conditions [294], of the highly elaborated anticancer drug vinorelbine (**45**). This provided in one step vinflunine (**46**) which, from 2009, became another anticancer drug. Past the historical case of fluoroquinolones [295], many more examples of the possible advantages of introducing fluorine(s) in a drug are provided in recent reviews [289,296]. Another example of a very late stage functionalization of molecule would be the use of zinc sulfinate chemistry [297] which, as depicted here, allowed to introduce in one step a trifluoromethyl group on the hydroquinidine **47** and access the otherwise rather hard-to-get compound **48**. In fact, CH functionalization is probably one the most studied reactions these days just for such reasons [298–300]. Moreover, these modern reactions do not diminish the potential interest of undertaking a good old halogenation of an active compound, under a variety of conditions, not only to reach new analogues but also to detect which parts are prone to oxidation/metabolization. A remarkable result, using diluted iodine, was actually reported recently [301].

**Figure 9:**
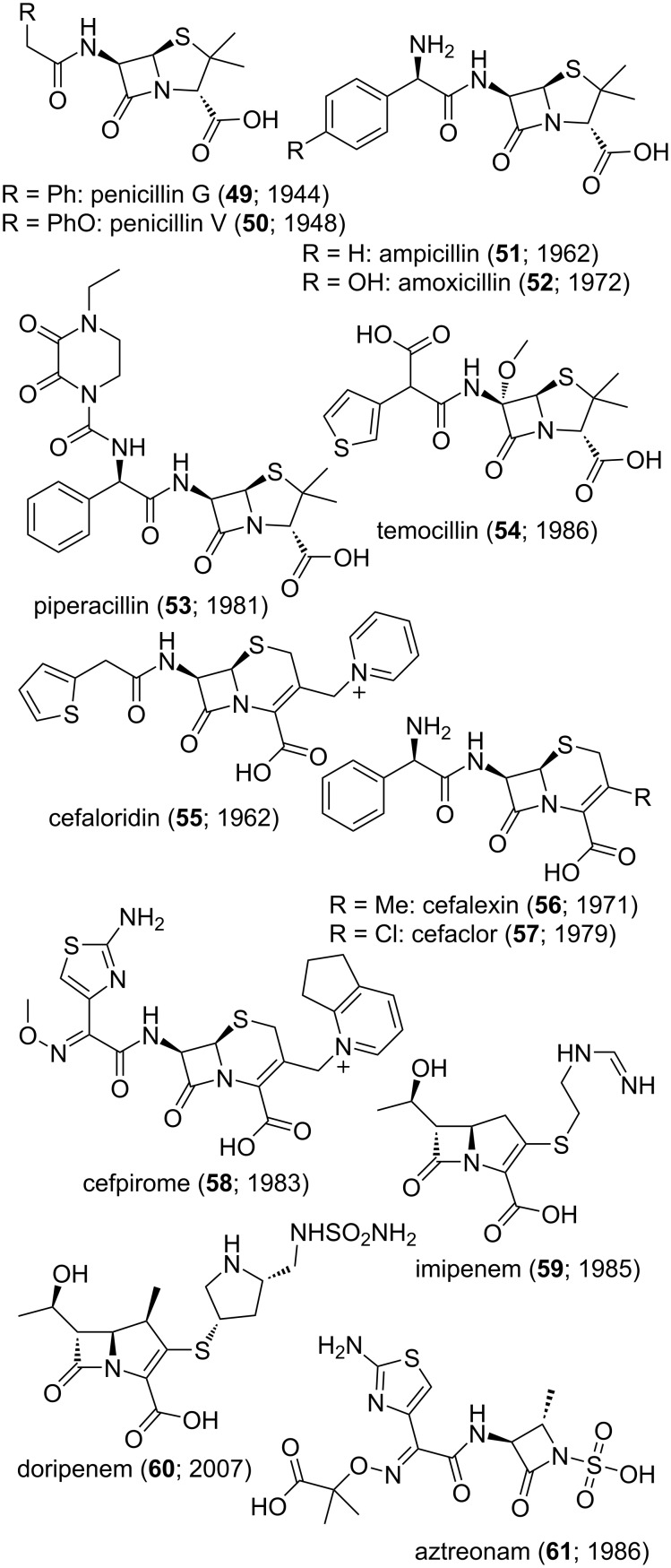
Structures of compounds **49–61**.

Concerning another class of “old” drugs, and as depicted in Figure 9 with the (few) structures **49**–**61**, the many generations of β-lactams used in human medicine is certainly one more illustration of how medicinal chemistry proceeds across decades of research. As well described in book chapters [302–303], these antibiotics owe their existence to the isolation of naturally occurring substances along with their (bio)transformations to improve their production and/or their human pharmacology. Moreover, preparing fully artificial β-lactam analogues is also an active research field since, for instance aztreonam (**61**), is produced by total synthesis [304–305]. Amongst other issues, the main concern in this domain remains the design of compounds capable of resisting the various bacterial β-lactamases but still reactive (and selective) enough to form relatively stable covalent adducts with the bacterial DD-transpeptidases. The carboxylic or sulfonic moieties in these structures are instrumental in this regard. As seen in Figure 9, the rate of new β-lactams approved for human use has slowed down but it has yet to stop. Could the use of very recent chemistry allow to go beyond the current [303,306–310] state of the art?

**Figure 10:**
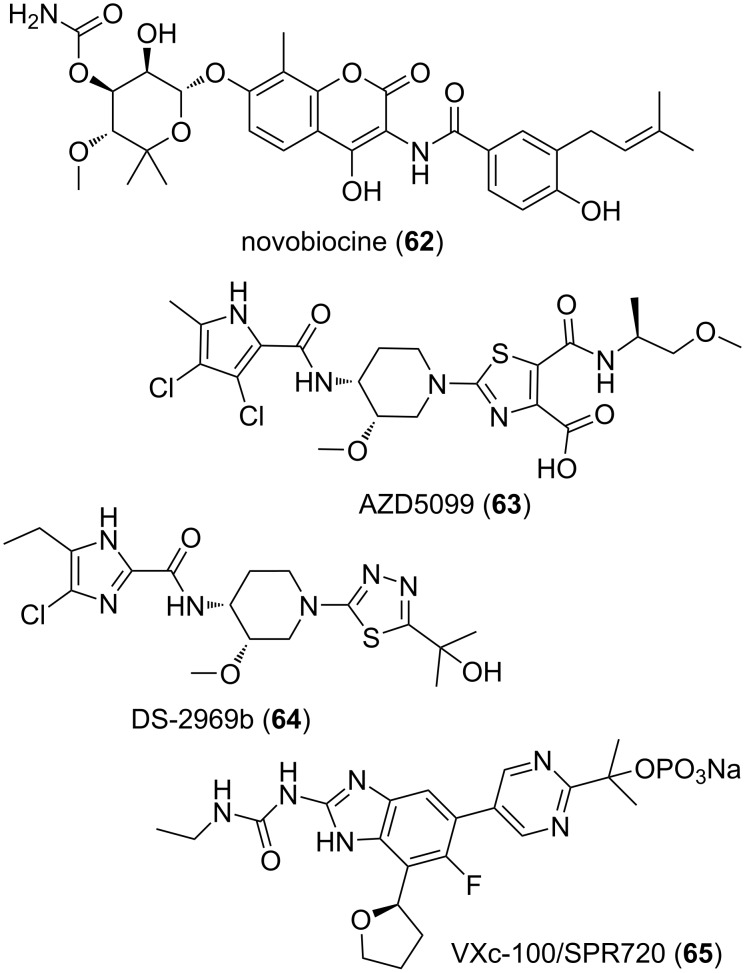
Structures of compounds **62**–**65**.

Aside from these rather thoroughly investigated β-lactam antibiotics (more than 40,000 tons are produced every year), one could consider the much less studied novobiocin (**62**) depicted in Figure 10. This compound is another naturally occurring antibiotic which was isolated in 1955 [311]. It turned out to be the first inhibitor of the bacterial ATP-ase function of gyrases found and it was actually instrumental in the discovery of these enzymes [312–313]. Since the ATP-ase function of these type IIA topoisomerases are not currently targeted by any prescribed antibiotics and since novobiocin (**62**) was banned in the past from humans use [314], it could be of interest to improve its very poor pharmacology. How about by removing its coumarone part? This is indeed a rather ”sticky” component [53] also found in frequent hitting substances and it could plausibly be at the source of some of its pharmacological problems. The choice of the chemical motifs to replace this part could benefit from the more recent wealth of structural and pharmacological data already available for this class of inhibitors such as compounds **63**–**65** [115,315]. Indeed, out of the structure–activity relationship of the many series of inhibitors of the ATP-ase function of type IIA topoisomerases discovered, it seems plausible to design and synthesize hybrids, combining the noviose moiety of novobiocin (**62**) and a heterocyclic component mimicking the one found in their structures. For instance, could it be possible to integrate the aminothiazole component of AZD5099 (**63**) [316], the aminothiadiazole component of DSP-2969b (**64**) [317–318], or the arylpyrimidine component of the phosphate prodrug [319–320] VXc-100/SPR720 (**65**)? Of note is that these three bacterial gyrase inhibitors have reached phase 1 clinical trials [321–324] although the evaluation of AZD5099 (**63**) was suspended in 2011 because of [315]: “(a) high variability in exposure within a small group of healthy volunteers […] (b) concerns related to mitochondrial changes observed in preclinical safety species”.

## Conclusion

**Figure 11:**
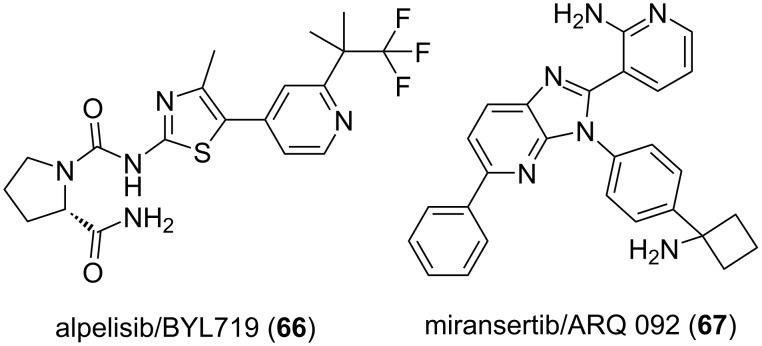
Structures of compounds **66** and **67**.

As probably not emphasized enough here, the discovery of drugs requires first a fair amount of luck to find a proper hit. It may then be that this requirement is what is at the source of the current medicinal chemistry recognition problem. In a world where predictability is an important factor for investors as well as grant calls, medicinal chemistry retains an embarrassing aspect of science which, to borrow from Louis Pasteur words on luck, requires to be ready to recognize the unexpected and then act on it. Despites decades of progress in computer science, it is still the constitution of large, diverse and well-maintained chemical libraries [325] to undertake well-designed screening campaigns which remains the way to spot the unexpected/unpredictable leading to a drug discovery. When considering the antibacterial world, the recent grant calls to mostly study microbial resistance maybe symptomatic of a loss of interest in academia for MedChem efforts plausibly because of a lack of potentially fruitful and original chemical libraries [197]. Moreover, a review on “the success and limitations” of strategies focusing on the bacterial “resistome” is of real interest [326]. Also noteworthy is a published roadmap for antibiotic discovery which, in 2021, had to emphasize the crucial role of medicinal chemistry [327]. The recently NIH-supported Chemistry Center for Combating Antibiotic-Resistant Bacteria (CC4CARB), which features an arm [328] focusing on the design of chemical libraries targeting Gram-negative bacteria, is a far more encouraging orientation in this regard. Additional oversea initiatives, focusing on other therapeutic areas, such as the national cancer institute NEXT program and the NINDS Blueprint Neurotherapeutic Network could also be adopted by other countries in order to sponsor their own academic research in organic/medicinal chemistry. Still on research funding agencies/organizations policy, project calls focusing on genetic-based diseases are usually not considering medicinal chemistry as a realistic approach. Hopefully, the kinase inhibitors depicted in Figure 11: alpelisib/BYL719 (**66**) [329] and miransertib/ARQ 092 (**67**) [330] which have demonstrated very promising results in clinical trials against one rare and terrible type of genetic-based pathology may alter this point of view in the future.

In any case, in addition to this plea, it is hoped that the overwhelming number of nonsensical investigations reported against COVID-19, which have been reviewed to some extent [331–338], should also be an intensive to improve the success rate of academic research in medicinal chemistry. Investing in medicinal chemistry, for instance to reach series of pertinent new chemical entities, remains a long-term strategy typical of public-funded research but the superiority of some alternative approaches developed in the past decades has yet to be demonstrated in the real world of drug discovery. If many authors (and referees) have withheld a judgment on these issues, it is hoped that this document triggers a long-deferred debate especially since too many academic decision-makers hold today the belief that a large computer is sufficient to discover a new drug. Even if the latest artificial intelligence-based drug discovery bid [339–341] delivers its many promises, the delays for a true demonstration could be, again, measured in decades [342]. As often stated ages ago by my Ph.D. supervisor: “it is quite all right to investigate and invest in new approaches in medicinal chemistry but not to the cost of abandoning the proven one”. Finally, the above should also provide a renewed demonstration that, whatever the selection process adopted [287,343], drug discovery is not only really benefiting from excellence in chemistry but that it is also at least on par with natural product total synthesis in terms of providing challenges, typical of academic research, in organic chemistry.
